# Identification of *Pseudomonas aeruginosa* exopolysaccharide Psl in biofilms using 3D OrbiSIMS

**DOI:** 10.1116/6.0002604

**Published:** 2023-05-31

**Authors:** Heba Khateb, Andrew L. Hook, Stefanie Kern, Julie A. Watts, Sonali Singh, Darryl Jackson, Luisa Marinez-Pomares, Paul Williams, Morgan R. Alexander

**Affiliations:** 1 Advanced Materials and Healthcare Technologies Division, School of Pharmacy, University of Nottingham, Nottingham, United Kingdom; 2 National Biofilms Innovation Centre, Biodiscovery Institute and School of Life Sciences, University of Nottingham, Nottingham, United Kingdom; 3 Nanoscale and Microscale Research Centre, University of Nottingham, Nottingham, United Kingdom; 4 National Biofilms Innovation Centre and School of Life Sciences, University of Nottingham, Nottingham, United Kingdom

## Abstract

Secondary ion mass spectrometry (SIMS) offers advantages over both liquid extraction mass spectrometry and matrix assisted laser desorption mass spectrometry in that it provides the direct *in situ* analysis of molecules and has the potential to preserve the 3D location of an analyte in a sample. Polysaccharides are recognized as challenging analytes in the mass spectrometry of liquids and are also difficult to identify and assign using SIMS. Psl is an exopolysaccharide produced by *Pseudomonas aeruginosa,* which plays a key role in biofilm formation and maturation. In this Letter, we describe the use of the OrbiTrap analyzer with SIMS (3D OrbiSIMS) for the label-free mass spectrometry of Psl, taking advantage of its high mass resolving power for accurate secondary ion assignment. We study a *P. aeruginosa* biofilm and compare it with purified Psl to enable the assignment of secondary ions specific to the Psl structure. This resulted in the identification of 17 peaks that could confidently be ascribed to Psl fragments within the biofilm matrix. The complementary approach of the following neutral loss sequences is also shown to identify multiple oligosaccharide fragments without the requirement of a biological reference sample.

## I. INTRODUCTION

Biofilms are communities of surface associated micro-organisms embedded in a self-generated extracellular matrix consisting of exopolysaccharides, proteins, and extracellular DNA (eDNA). The biofilm matrix facilitates survival in hostile, shifting environments and protects cells from the stresses imposed by nutrient limitation, desiccation, antimicrobial agents, and host defense mechanisms.^1–4^ The multiantibiotic resistant human bacterial pathogen *Pseudomonas aeruginosa* produces three major exopolysaccharides (alginate, Pel, and Psl) that contribute to the biofilm matrix.^2,5,6^ Alginate, which consists of β-1,4 linked l-guluronic and mannuronic acid, is predominately generated by mucoid clinical isolates of *P. aeruginosa* associated with chronic lung infections in individuals with cystic fibrosis.^7^ Nonmucoid strains can use either Pel or Psl as the primary matrix structural exopolysaccharide. Pel is a cationic exopolysaccharide, consisting of a partially de-*N*-acetylated linear polymer of α-1,4-*N*-acetylgalactosamine comprised predominantly of dimeric repeats of galactosamine and *N*-acetylgalactosamine.^8^ Pel can cross-link eDNA within the *P. aeruginosa* biofilm matrix providing additional structural integrity.^2,9,10^ The third *P. aeruginosa* exopolysaccharide, Psl, is implicated in promoting surface adhesion, maintaining biofilm architecture via cell–cell and cell–surface interactions, and in signaling.^11,12^ During migration over a surface, certain *P. aeruginosa* strains lay Psl trails leading to biofilm initiation.^13^ Psl also acts as a signal molecule enhancing biofilm formation by increasing the production of Psl and other biofilm components.^14^ Nuclear magnetic resonance analyses have established that Psl consists of a repeating pentasaccharide unit consisting of three d-mannose, an l-rhamnose, and a d-glucose.^11^

Polysaccharides such as Psl are difficult to identify and assign using mass spectrometry methodologies, including SIMS.^15–18^ Recently, a label-free molecular imaging technique, 3D OrbiSIMS, has been developed that employs both a hybrid instrument with a time-of-flight (ToF) mass spectrometer for high speed 3D imaging and a high-field Orbitrap MS analyzer with a high mass resolving power that functions at a slower speed. OrbiSIMS, offers micrometer scale molecular spatial resolution with a high mass resolution (>240 000 at m/z 200) enabling far more accurate peak assignment than ToF SIMS.^19,20^ By using an instrument equipped with cryogenic sample handling and high-pressure freezing protocols, cryo-OrbiSIMS can be used for high-resolution MS imaging of biological samples preserved in the frozen-hydrated state, the closest to their native state that can be achieved in vacuum. Zhang *et al*. previously acquired positive ion data from a cryo-OrbiSIMS depth profile of a mature frozen-hydrated *P. aeruginosa* biofilm.^20^ A targeted approach was used for peak assignment in this data set, which revealed 2-alkyl-4-quinolones and their related *N*-oxides (a total of 33), *N*-acyl homoserine lactones (6), rhamnolipids (7), nucleobases (5), amino acids (20), lipids (15), and miscellaneous metabolites, with a mass deviation < 2 ppm.

Loss analysis has been shown to be an efficient way of annotating mass spectra in tandem MS data derived from soft ionization sources such as electron spray ionization.^21–23^ It has also been used for annotating predictable and unpredictable metabolic features in untargeted metabolomics.^24^ Recently, this approach was applied to the OrbiSIMS analysis of proteins for the first time.^25^ The relatively *hard* ionization of SIMS produced sufficient fragmentation to enable neutral loss analysis, whereupon various peptide sequences were identified within protein samples.

In this Letter, we describe the targeted identification of Psl peaks in cryo-OrbiSIMS data from a frozen wild type *P. aeruginosa* PAO1 biofilm (Pel^+^/Psl^+^) published previously^20^ using a purified exopolysaccharide sample (low molecular weight; < 45 kDa) prepared from a Psl-overproducing *P. aeruginosa* strain Δ*wspF* Δ*pel* (Pel^−^/Psl^+^)^26^ as described by Watanabe and Makino.^27^ To facilitate this process, a negative Psl control was employed, which was a frozen-hydrated biofilm formed by a *P. aeruginosa* PAO1 Δ*wspF* Δ*psl* mutant (Pel^+^/Psl^−^) that cannot produce Psl. The classification of neutral losses in the mass spectra was used to identify putative Psl fragment ions. These findings will be essential for future studies on *P. aeruginosa* biofilms and their molecular precursors at surfaces.

## II. EXPERIMENT

### A. Bacterial strains and growth condition

*P. aeruginosa* PAO1 Δ*wspF* Δ*psl* (Pel^+^/Psl^−^) biofilms were grown in RPMI-1640 (without l-glutamine or phenol red) (Lonza) with 0.02 concertation at OD_600_. Biofilm was directly grown on the surface at 37 °C for 24h. After growth, biofilms were washed 2–3 times with 150 mM ammonium formate solution and assembled in a sample carrier system for high pressure freezing as described by Zhang *et al*.^20^ Before measurement by cryo-OrbiSIMS, samples were placed in a cryomanipulation station, from where they were transferred to the cryo-OrbiSIMS using a shuttle chamber.^20^

To reduce the salt content, the samples were washed with ammonium formate (150 mM), wicking off excess liquid and plunging into liquid nitrogen to cryoimmobilize the system. They were maintained under liquid nitrogen during transfer to a Leica VCM, manually clamped onto a sample holder, and transferred to the hybrid SIMS instrument using a Leica VCM and VCT system, maintaining liquid nitrogen conditions. SIMS analysis was performed at or below −160 °C.

### B. Psl Purification

Psl-rich exopolysaccharides were extracted and purified from *P. aeruginosa* PAO1 Δ*wspF* Δ*pel* (Pel^−^/Psl^+^) via cold ethanol precipitation and size exclusion chromatography as described before.^28^ A 1 mg/ml Psl solution was applied to a coverslip for 10 min prior and allowed to dry before OrbiSIMS analysis.

### C. OrbiSIMS analysis

The *P. aeruginosa* PAO1 (Pel^+^/Psl^+^) biofilm data were taken from the literature.^20^ OrbiSIMS analyses of purified Psl and frozen-hydrated Psl^−^ mutant *P. aeruginosa* PAO1 Δ*wspF* Δ*psl* (Pel^+^/Psl^−^) biofilms were carried out as described previously.^20^ A 20 keV Ar_3000_^+^ analysis beam of 20 *μ*m diameter was used as the primary ion beam. The gas cluster ion beam (GCIB) duty cycle was set to 4.4%, with a current of 218 pA. The depth profile was run on an area of 200 × 200 *μ*m using a random raster mode with a crater size of 284.5 × 284.5 *μ*m. The cycle time was set to 400 *μ*s. The optimal target potential was approximately +68 V. Argon gas flooding was in operation to aid charge compensation and pressure in the main chamber was maintained at 9.0 × 10^−7^ mbar. The spectra were collected in positive polarity in the *m/z* range of 150–2250. The injection time was set at 500 ms. 100 and 200 scans were run for the purified Psl and bacterial biofilm, respectively. Mass resolving power was set to 240 000 at *m/z *= 200.

### D. Data processing

Peak assignment and neutral loss analysis was conducted using a custom built VBA (Visual Basic for Application) script.^29^ Possible assignments were limited to a deviation of no more than ±3.5 ppm, a maximum of 20 double bonds equivalence, and a C:O ratio from 0.5 to 1.5. Ions with possible saccharide assignments were then manually inspected to discount noise and convoluted peaks, and MS images were used to confirm that the selected peaks were not noise.

## III. RESULTS AND DISCUSSION

### A. Identification of Psl in biofilms and purified Psl samples

**FIG. 1.**
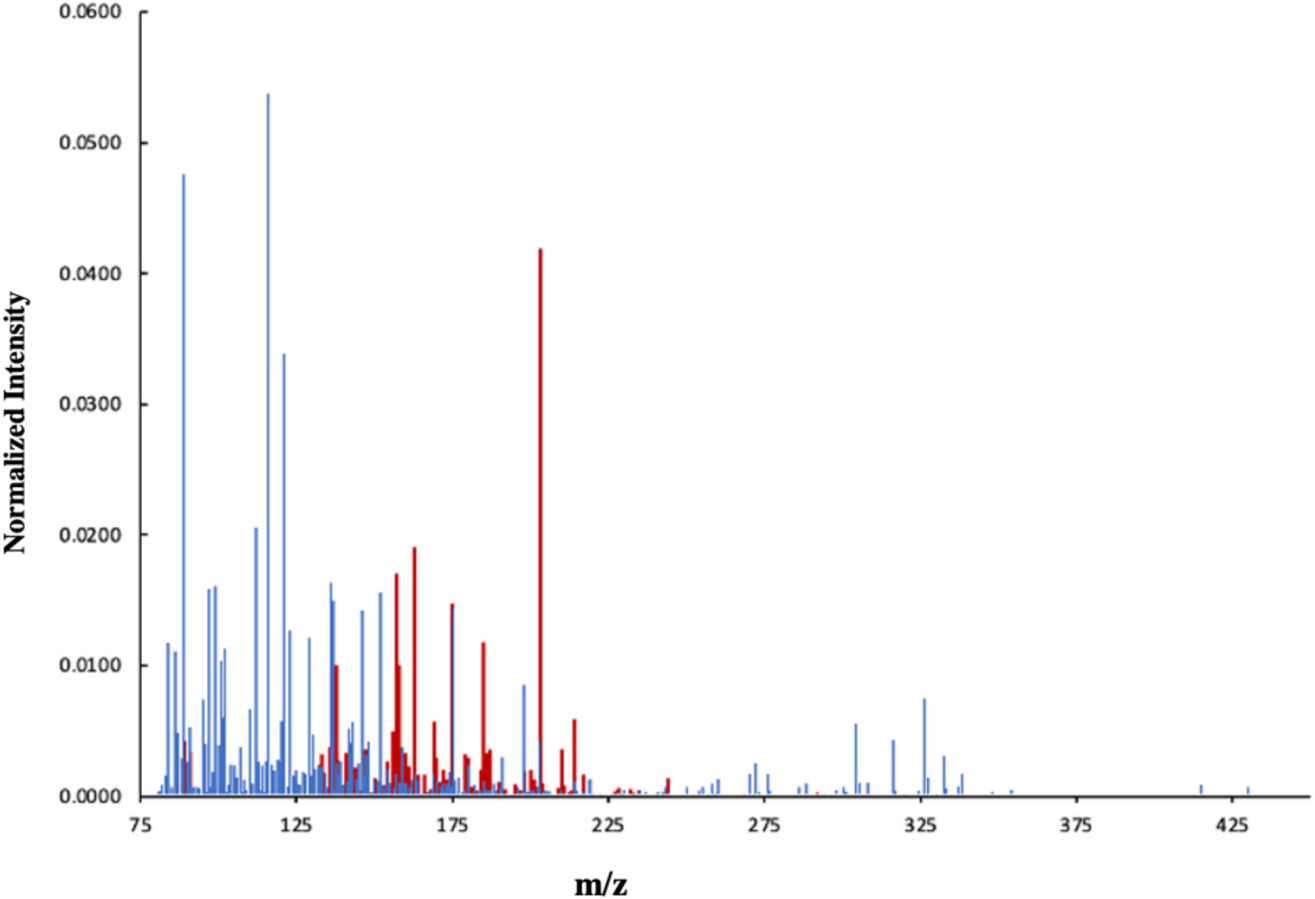
Positive ion 3D OrbiSIMS spectra of the frozen-hydrated *P. aeruginosa* PAO1 (Pel^+^/Psl^+^) biofilm represented in blue with the saccharide fragments in red. The saccharide fragment intensity was multiplied by ×10 for easier visualization, spectral data acquired from Ref. 20.

Initially, the highest intensity secondary ions derived from the published positive OrbiSIMS spectra of *P. aeruginosa* PAO1 (Pel^+^/Psl^+^) biofilms were analyzed^11,19^ and the possible saccharide ions were assigned within an accurate mass assigned composition range of C_6−100_H_6−100_O_1−100_ K/Na_0−100_ with a maximum number of double bonds = 20, rings size = 5, maximum unpaired values = 20 with deviation <3.5, which are listed in Fig. 1 and Table S1.^34^ In total, 223 possible saccharide ions were identified in the PAO1 biofilm, representing 2.5% of the total number of positive polarity peaks identified from an automated peak search when only peaks with intensities over 2000 counts were considered between m/z 75 and 459 Da. Chemical images of example ions are presented in Fig. S1A,^34^ which are of low intensity and do not offer any useful insights into the distribution of chemistry within the biofilm in this case since they appear to be dominated by the biofilm topography suggested by the total ion intensity.

**FIG. 2.**
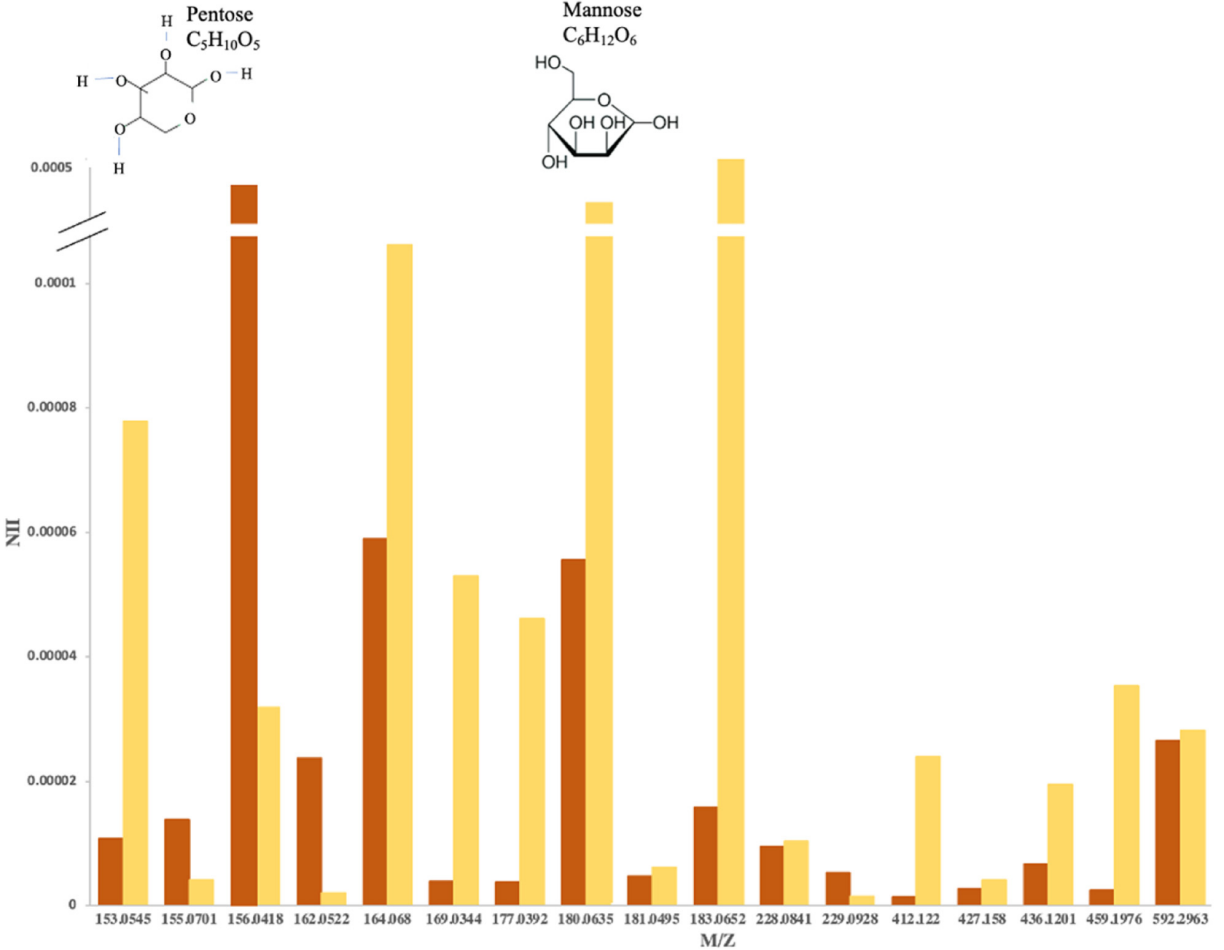
Most intense saccharides found in the 3D OrbiSIMS spectra from the frozen-hydrated *P. aeruginosa* PAO1 (Pel^+^/Psl^+^) biofilm (orange) and the purified Psl 1** **mg/ml (yellow).

The same OrbiSIMS analysis was undertaken for the purified Psl sample^28^ and the *P. aeruginosa* Δ*wsp*F Δ*psl* (Pel^+^/Psl^−^) biofilm. A total of 223 peaks were assigned to Psl-related peaks based on the comparison of the acquired OrbiSIMS spectra for the purified Psl with that of the *P. aeruginosa* Psl-negative biofilm. Of these, 30 peaks (Table S2)^34^ were common to both the purified Psl and PAO1 (Pel^+^/Psl^+^) biofilm. There were 14 of these that were also present in the Psl-negative biofilm of *P. aeruginosa* Δ*wsp*F Δ*psl* (Pel^+^/Psl^−^) (Table S3) (Fig. S2),^34^ leaving the remaining 17 peaks common to the purified Psl and the PAO1 (Pel^+^/Psl^+^) biofilm as Psl peaks (Fig. 2 and Table S4).^34^

By virtue of their selection process, none of the 17 peaks were detected in the biofilm formed by the negative control *P. aeruginosa* (Pel^+^/Psl^−^). Psl has a pentasaccharide repeating unit, and since polysaccharides can produce ions by cleaving glycosidic bonds and fragmenting cross-rings, the assignment of Psl was based on the mono-, di-, tri-, and tetra-saccharide fragments that form the repeat unit. The remaining 206/223 ions that could potentially be assigned to saccharides were concluded to not be specific to Psl but could come from the fragmentation of other molecules.

The Psl pentasaccharide repeat unit consists of three d-mannose, one l-rhamnose, and one d-glucose molecules.^3,11^ The major components, mannose/glucose, which is repeated three times, and rhamnose, were identified in the positive ion spectra at m/z 180.0635^30^ and 164.068,^27,31^ in both the Psl^+^ biofilm and the purified Psl with high intensity, while neither were found in the acquired OrbiSIMS data of the *P. aeruginosa* Δ*wspF* Δ*psl* mutant (Pel^+^/Psl^−^).

The 17 Psl-associated ions were classified into 12 possible putative structures identified as monosaccharide peaks in addition to three possible trisaccharide intensities shown in Fig. 2, namely, C_15_H_24_O_13_^ +^, C_18_H_30_O_14_^ +^, and C_17_H_24_O_13_^ +^ at 412.122, 427.158, and 436.1201 m/z, respectively, and two possible tetrasaccharide peaks, NaC_23_H_32_O_8_^ +^, and C_24_H_48_O_16_^ +^ at 459.1976 m/z at 592.2963 m/z, respectively. The monosaccharide (C_5_H_10_O_5_^+^) at 153.0545 m/z showed the highest intensity for the PAO1 (Pel^+^/Psl^+^) biofilm, the monosaccharide (C_9_H_11_O_4_^ +^) at 183.0652 m/z was the most intense for the purified Psl, whereas the trisaccharide (C_24_H_48_O_16_^ +^) at 592.2963 m/z showed an equal intensity for both the PAO1 and purified Psl. Chemical images are presented in Fig. S1B,^34^ showing similar low intensity high noise maps following the total ion intensity distribution.

### B. Neutral loss analysis

**FIG. 3.**
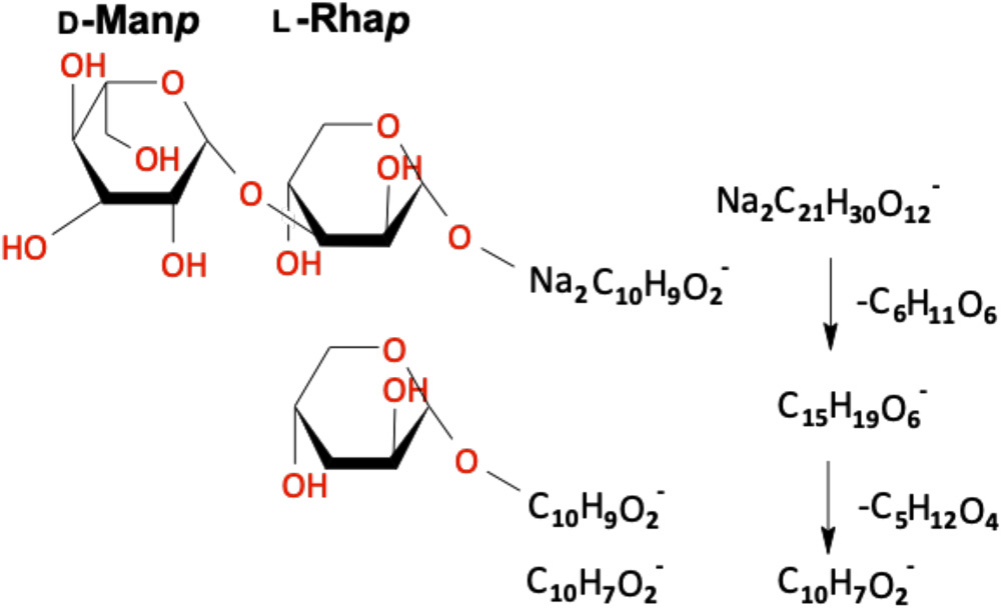
Possible chemical structure of a Psl trisaccharide identified using neutral loss analysis. Possible structures for the sequence of sugars are also shown. Assignment of specific sugars is based upon the previously determined Psl penta-saccharide (Ref. 11); however, mannose and glucose units could not be differentiated by the 3D OrbiSIMS analysis and the composition of the final daughter ion could not be determined.

As an alternative approach to identify Psl fragments, we performed a neutral loss analysis^32,33^ on the *P. aeruginosa* PAO1 biofilm data. This approach has previously been utilized for *de novo* peptide sequencing using 3D OrbiSIMS data.^25^ The data were screened to identify neutral losses between ions that corresponded to C_5_ or C_6_ sugar units. In total, 1168 different ion pairs from 1713 different ions were identified with a possible single sugar loss between them. This included the ion at 459.1976 m/z (NaC_23_H_32_O_8_^ +^) identified as derived from Psl from the analysis of the *P. aeruginosa* ΔwspF Δpsl mutant (Pel^+^/Psl^−^). Multiple pairs featured the same mother ion with different daughter ions. In 16 cases, neutral loss sequences were identified featuring three or more ions, allowing for the identification of saccharide sequences. The possible structure of one sequence with the smallest daughter ion is shown in Fig. 3. There was oxygen depletion in the daughter ion compared to the C:O ratio of a sugar unit, suggesting the loss of labile hydroxyl groups. Moreover, as the number of carbon atoms is > 6, the daughter ion must be derived from two sugar units. The daughter unit could, thus, not be assigned to a specific pair of sugars, but likely does originate from a disaccharide due to associated neutral loss fragments corresponding to sugar units. The first neutral loss fragments were consistent with intact C_6_ sugars and could be assigned to either mannose or glucose. The second neutral loss fragment was oxygen and carbon depleted compared to an intact sugar unit but may be associated with rhamnose considering the low oxygen number. The presence of a rhamnose–glucose or rhamnose–mannose disaccharide within Psl is consistent with the previously reported Psl pentasaccharide.^11^ Thus, the assessment of neutral loss fragments allowed for the *de novo* assignment of oligosaccharide fragments; however, establishing saccharide sequence is limited by (1) structural/stereo isomers that cannot be differentiated by the OrbiTrap mass analyzer and (2) loss of labile hydroxyl groups. Nevertheless, this methodology enables the confident assignment of 3D OrbiSIMS ions to specific polysaccharides without the need for biological reference samples and could also be applied to detect ions associated with other biopolymers.

In conclusion, the OrbiSIMS data from the *P. aeruginosa* PAO1 biofilm (Pel^+^/Psl^−^),^20^ purified Psl, and a *P. aeruginosa* (Pel^+^/Psl^−^) mutant biofilm were all analyzed and compared to identify the ion characteristic of Psl. Initially, a stoichiometric filtering of the secondary ion peaks was employed for the *P. aeruginosa* biofilm, resulting in an assignment of 223 possible saccharide ions, contributing approximately 2.5% of the total biofilm ions. Of these ions, 17 ion peaks were observed in the purified Psl and PAO1 biofilm but not in the PAO1 Psl negative mutant. As an alternative approach, neutral loss analysis identified an ion sequence associated with a pentasaccharide that could confidently be assigned to Psl without the requirement of a biological reference sample, but requires prior knowledge of the presence of a polysaccharide. These indicative ions are expected to be of use in future studies where the role of Psl in surface sensing and adhesion is studied.

## Data Availability

The data that support the findings of this study are available within the article and its supplementary material.
